# A nationwide cohort study suggests clarithromycin-based therapy for *Helicobacter pylori* eradication is safe in patients with stable coronary heart disease and subsequent peptic ulcer disease

**DOI:** 10.1186/s12876-022-02498-1

**Published:** 2022-09-12

**Authors:** Yen-Chun Chen, Yi-Da Li, Ben-Hui Yu, Yi-Chun Chen

**Affiliations:** 1 Division of Hepato-Gastroenterology, Department of Internal Medicine, Dalin Tzu Chi Hospital, Buddhist Tzu Chi Medical Foundation, Chiayi, Taiwan; 2 School of Medicine, Tzu Chi University, Hualien, Taiwan; 3 Division of Cardiology, Department of Internal Medicine, Dalin Tzu Chi Hospital, Buddhist Tzu Chi Medical Foundation, Chiayi, Taiwan; 4 Department of Radiation Oncology, Dalin Tzu Chi Hospital, Buddhist Tzu Chi Medical Foundation, Chiayi, Taiwan; 5 Division of Nephrology, Department of Internal Medicine, Dalin Tzu Chi Hospital, Buddhist Tzu Chi Medical Foundation, No. 2, Minsheng Rd., Dalin Township, Chiayi, Chiayi County 622 Taiwan

**Keywords:** Clarithromycin, Overall mortality, Cardiovascular mortality, Cardiovascular morbidity, Peptic ulcer disease, *Helicobacter pylori*

## Abstract

**Background:**

Clarithromycin-based therapy is important for *Helicobacter pylori* eradication treatment. However, clarithromycin may increase cardiovascular risk. Hence, we investigated the association between clarithromycin use and outcomes in adults with stable coronary heart disease (CHD) and subsequent peptic ulcer disease (PUD).

**Methods:**

This nationwide cohort study used a national health insurance database to screen 298,417 Taiwanese residents who were diagnosed with coronary heart disease from 2001 to 2015 for eligibility in the study and to evaluate select eligible patients with CHD–PUD from 2004 to 2015. Data were obtained from new users of clarithromycin (n = 4183) and nonusers of clarithromycin (n = 24,752) during follow-up. A total of 4070 eligible clarithromycin users and 4070 nonusers were subject to final analysis by 1:1 propensity score matching. Participants were followed up after receiving clarithromycin or at the corresponding date until the occurrence of cardiovascular morbidity in the presence of competing mortality, overall mortality and cardiovascular mortality, or through the end of 2015. The incidence rates and risks of overall mortality and cardiovascular outcomes were evaluated. The associations between clarithromycin and arrhythmia risk, as well as its dose and duration and overall mortality and cardiovascular outcomes were also addressed.

**Results:**

Clarithromycin users were associated with adjusted hazard ratios of 1.08 (95% confidence interval, 0.93–1.24; 21.5 compared with 21.2 per 1000 patient-years) for overall mortality, 0.95 (0.57–1.59; 1.5 compared with 1.8 per 1000 patient-years) for cardiovascular mortality, and 0.94 (0.89–1.09; 19.6 compared with 20.2 per 1000 patient-years) for cardiovascular morbidity in the presence of competing mortality, as compared with nonusers. We found no relationship between dose and duration of clarithromycin and overall mortality and cardiovascular outcomes and no increased risk of arrhythmia during follow-up period. After inclusion of arrhythmia events to re-estimate the risks of all study outcomes, the results remained insignificant.

**Conclusion:**

Concerning overall mortality, cardiovascular mortality, and cardiovascular morbidity, our results suggest clarithromycin-based therapy for *Helicobacter pylori* eradication may be safe in patients with stable CHD and subsequent PUD.

**Supplementary Information:**

The online version contains supplementary material available at 10.1186/s12876-022-02498-1.

## Introduction

*Helicobacter pylori* (*H. pylori*) infection has been identified as a risk factor for several gastric and extra-gastric diseases such as gastric cancer, coronary heart disease (CHD), ischemic stroke [1], iron deficiency anemia [2], and non-alcoholic fatty liver disease [3]. The overall prevalence of *H. pylori* infection is high, estimated to be more than half of the world's population [4]. However, its incidence varies in different regions and is higher in those with lower socioeconomic status. Most patients with peptic ulcers are *H. pylori*-infected [5, 6]. *H. pylori* eradication can prevent the recurrence of gastric and duodenal ulcers [7]. Furthermore, *H. pylori* infection can increase the risk of peptic ulcer bleeding, especially in those taking antiplatelet agents, including aspirin, non-aspirin antiplatelet agents, or combined antiplatelet therapy [8]. For patients with cardiovascular disease, secondary prevention by antiplatelet agents is essential. *H. pylori* is also linked to atherosclerosis and ischemic heart disease [9], and *H. pylori* eradication decreased CHD risk in some patients with peptic ulcers compared with those not receiving *H. pylori* eradication therapy [10]. Therefore, eradication therapy may be essential for patients with cardiovascular disease who are also infected with *H. pylori*. Previous studies have aimed to find alternative medication for secondary prevention in addition to antiplatelet therapy [11–13]. They investigated the role of macrolide antibiotics for patients with CHD in addition to antiplatelet treatment [11–13]. One study found that roxithromycin, a kind of macrolide class, may benefit patients with CHD [11]. Clarithromycin is another kind of macrolide antibiotic. A randomized, double-blind study investigated patients with unstable heart disease treated with clarithromycin or placebo for 3 months. The authors found that clarithromycin users had decreased cardiovascular events [12]. Later, another CLARICOR trial [13] examining the effects of clarithromycin on mortality and morbidity in patients with ischemic heart disease was conducted to evaluate the impact of short-term use of clarithromycin among those with stable heart disease. That trial showed that clarithromycin users had a higher cardiovascular mortality rate within 3 years. Another study evaluated cardiovascular risk after clarithromycin use [14] among patients who were diagnosed with chronic obstructive pulmonary disease (COPD) with acute exacerbations and community-acquired pneumonia. The study revealed a harmful effect of clarithromycin. However, COPD with acute exacerbations and community-acquired pneumonia was associated with ischemic heart disease or cardiac complications [15–17]. Other observational studies investigated the risk of clarithromycin for cardiac events among patients with acute infectious disease. These observational studies also showed conflicting results [18, 19].

In 2018, the US Food and Drug Administration warned about possible cardiovascular events or death from clarithromycin among patients with CHD [20]. Though the resistance rate of clarithromycin is higher currently than in the past, clarithromycin is still a crucial antimicrobial in the combination therapy for *H. pylori*, especially in areas where the resistance rate is less than 15% [21, 22]. Therefore, it is essential to clarify the risk of cardiovascular mortality and morbidity after clarithromycin use. We analyzed a national healthcare database to investigate whether clarithromycin treatment for *H. pylori* was associated with increased risk for overall mortality and cardiovascular outcomes among adults with stable CHD and subsequent PUD.

## Materials and methods

### Data source

This retrospective nationwide cohort study used outpatient and inpatient claims data from Taiwan's 2005 Longitudinal Generation Tracking Database (LGTD2005) between January 1, 2000 and December 31, 2015. The LGTD2005 has been described in detail in our previous research [23]. In brief, it is a de-identified database released by the Health and Welfare Data Science Center of the Taiwan Ministry of Health and Welfare for research purposes. Thus, this study did not require informed consent and was exempt from full review by the Institutional Review Board of the Dalin Tzu Chi Hospital (B10702014 and B11101016). The database included 2 million people who were randomly sampled in the year 2005 from all beneficiaries in Taiwan's compulsory universal National Health Insurance (NHI) program with > 99% coverage [24] and tracked all medical records from 2000 until 2015. It has been documented that there was no significant difference in age, gender, region, ambulatory care, and inpatient expenditures between the LGTD2005 and the NHI program. The LGTD2005 contains comprehensive medical information, except laboratory and lifestyle data, and adopts ICD-9-CM diagnosis codes to define diseases and anatomical therapeutic chemical codes to identify drugs.

### Study population (patients with stable CHD and subsequent PUD[CHD–PUD])

**Fig. 1:**
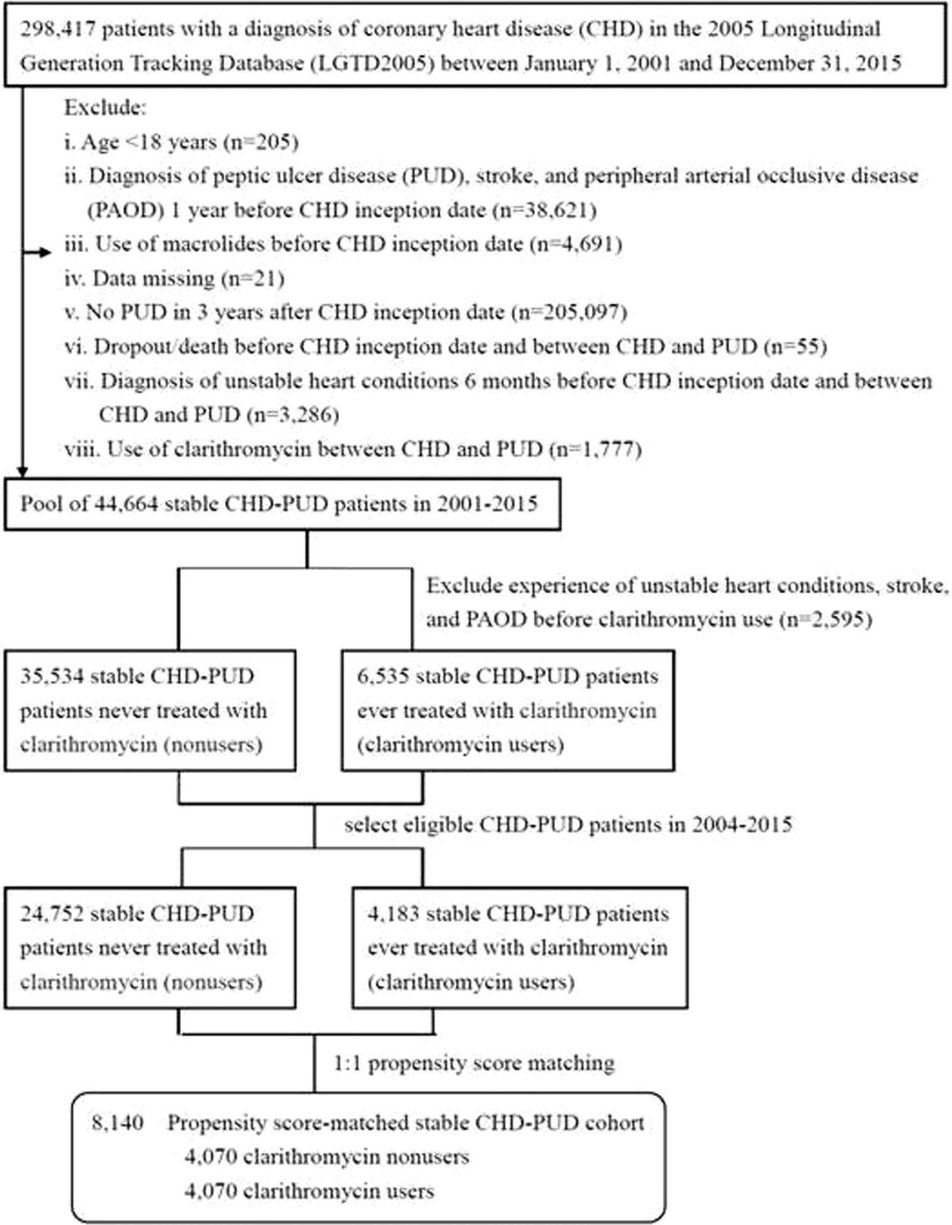
Study flowchart of patient selection

We identified 298,417 patients who had a diagnosis of CHD (ICD-9-CM codes 410–414) [25, 26] between January 1, 2001, and December 31, 2015 (Fig. 1). We excluded patients who were aged < 18 years; diagnosed with PUD (ICD-9-CM codes 531–534) [27], stroke, and peripheral arterial occlusive disease (PAOD) 1 year before CHD inception date; treated with macrolides before CHD inception date; subject to missing data; not diagnosed with PUD in the 3 years after CHD inception date; diagnosed with unstable heart conditions 6 months before CHD inception date and between CHD and subsequent PUD (CHD–PUD); considered to have dropped-out or died before CHD inception date and between CHD–PUD, and treated with clarithromycin between CHD–PUD. Patients with unstable heart conditions were defined as those who had ever been admitted to cardiovascular service due to a principle diagnosis of CHD or underwent three cardiac fluoroscopic interventions [28], including cardiac angiography with or without percutaneous transluminal coronary angioplasty, and cardiac electrophysiological study. Thus, in 2001–2015, the final pool was 44,664 patients with stable CHD–PUD, namely, who experienced no unstable heart conditions 6 months before CHD inception date and between CHD–PUD. To prevent immortal bias, we used the incident user (exposed to clarithromycin) design. We divided the final pool into two groups by the first prescription of clarithromycin: 33,534 nonusers (patients who never used clarithromycin throughout the study period) and 6535 clarithromycin users (patients who had been treated with clarithromycin at any time throughout the study period and who had experienced no unstable heart conditions, stroke, and PAOD before clarithromycin use). For avoiding survival bias, we selected eligible CHD–PUD patients from 2004 to 2015 and included 24,752 nonusers and 4183 clarithromycin users. Each clarithromycin user was propensity-matched with one nonuser, and the baseline for matching was set at the day when clarithromycin commenced in the user group and the corresponding date in the nonuser group. The propensity score was calculated using the logistic regression that was built on all baseline covariates listed in Table 1 to adjust for the baseline differences between clarithromycin users and nonusers. The propensity score model was reliable (Hosmer–Lemeshow test *p* = 0.91) and provided fair discrimination between the two groups (c-index, 0.62). A total of 8140 patients with stable CHD–PUD were subjected to the final analysis.

**Table 1:** Characteristics of study cohort by the use of clarithromycin

Variable	Propensity score-matched patients with stable CHD–PUD (n = 8140)		*p value*
	Clarithromycin users	Nonusers	
	(n = 4070) N (%)	(n = 4070) N (%)	
Sex			0.07
Men	2008 (49.3)	1926 (47.3)	
Women	2062 (50.7)	2144 (52.7)	
Age (year)			0.79
18–49	1263 (31.0)	1289 (31.7)	
50–59	1289 (31.7)	1248 (30.7)	
60–69	889 (21.8)	892 (21.9)	
≥ 70	629 (15.5)	641 (15.8)	
Mean (± SD)	55.8 ± 12.8	55.5 ± 13.9	0.46
Comorbidity			
Diabetes	703 (17.3)	603 (14.8)	0.003
Hypertension	1812 (44.5)	1724 (42.4)	0.049
COPD	723 (17.8)	679 (16.7)	0.20
Charlson comorbidity index (mean ± SD)	1.0 ± 1.5	1.0 ± 1.6	0.08
No. of medical visits			0.82
1–12	1349 (33.1)	1374 (33.8)	
13–24	1343 (33.0)	1339 (32.9)	
≥ 25	1378 (33.9)	1357 (33.3)	
Confounding drugs			
ACEI/ARB	956 (23.5)	900 (22.1)	0.14
Aspirin	1253 (30.8)	1141 (28.0)	0.006
Statins	499 (12.3)	466 (11.5)	0.26
Ticlopidine	23 (0.6)	26 (0.6)	0.67
Calcium channel blockers	1483 (36.4)	1458 (35.8)	0.56
Beta blockers	841 (20.7)	793 (19.5)	0.18
Diuretics	1903 (46.8)	1823 (44.8)	0.08
Antiarrhythmics	1068 (26.2)	1059 (26.0)	0.82
Digoxin	81 (2.0)	79 (1.9)	0.87
Nitrates	847 (20.8)	802 (19.7)	0.21

*CHD* coronary heart disease, *PUD* peptic ulcer disease, *COPD* chronic obstructive pulmonary disease, *SD* standard deviation, *ACEI/ARB* angiotensin-converting-enzyme inhibitor/angiotensin II receptor blocker

### Potential confounders

In addition to age and sex, the baseline comorbidities, including diabetes, hypertension, and COPD, were identified within 1 year before CHD diagnosis. The Charlson comorbidity index (CCI) was used to control for confounding factors [29], and the number of medical visits was used to minimize detection bias [24, 30] in studies using administrative databases. The confounding drugs were also identified throughout the study period and included angiotensin-converting enzyme inhibitor/angiotensin II receptor antagonist (ACEI/ARB), aspirin, ticlopidine, calcium channel blockers, beta-blockers, nitrates, diuretics, antiarrhythmics, and digoxin.

### Definition and ascertainment of study outcomes

The primary study outcome was overall mortality. The secondary study outcome was cardiovascular mortality, defined as death due to CHD, stroke, and PAOD. The tertiary study outcome was cardiovascular morbidity, which was defined as the experience of the above-mentioned unstable heart conditions, CHD, stroke, and PAOD. Death was defined as the withdrawal of the patients from the NHI program [31]. The clarithromycin users were followed up after administration of clarithromycin, whereas nonusers were followed up after the matched corresponding date [23]. All participants were observed for the occurrence of the primary, secondary, and tertiary study outcomes, or until the end of the study (December 31, 2015), whichever came first.

### Statistical analysis

We presented and compared baseline characteristics between clarithromycin users and nonusers based on the *t* test for continuous variables and the chi-square test for categorical variables. We calculated the incidence rates per 1000 person-years of the three study outcomes in the two groups. After confirming the assumption of proportional hazards by plotting the graph of the survival function versus the survival time and the graph of the log (-log(survival)) versus the log of survival time, we applied the Cox proportional hazard model to examine the association of clarithromycin with overall mortality and cardiovascular mortality after adjustments for age by year, sex, comorbidities, CCI, the number of medical visits, and 10 confounding drugs, and applied competing risk analysis [32] to examine the association of clarithromycin with cardiovascular morbidity. Moreover, we calculated each patient's cumulative defined daily dose (cDDD) of clarithromycin recommended by the WHO [33] to address the dose–response relationship of clarithromycin with study outcomes, given the usual daily dose of clarithromycin is 1 g and isometric increase in cDDD is 2000. We also evaluated the duration (≦ 7 days vs. > 7 days) [22] of clarithromycin prescription with study outcomes and analyzed the association between clarithromycin and the risk of arrhythmia (ICD-9-CM codes 427.x, 427.xx). To assess the reliability of our main findings, we conducted a sensitivity analysis to include arrhythmia events to re-estimate the risks of overall mortality and cardiovascular outcomes. All data were analyzed using SAS (version 9.4; SAS Institute, Inc., Cary, N.C.). A two-sided *p* value less than 0.05 was considered statistically significant.

## Results

### Patient characteristics

Among 4183 clarithromycin users, 4070 were propensity score-matched to a control set of 4070 nonusers at a 1:1 ratio (Fig. 1). The average age of both groups was 55 years, and the CCI was 1.0 (Table 1). The proportions of medical visits and patients with COPD and those receiving ACEI/ARB, ticlopidine, calcium channel blockers, beta-blockers, nitrates, diuretics, antiarrhythmics, and digoxin were similar within both groups. Clarithromycin users had a higher proportion of diabetes, hypertension, and aspirin prescription than nonusers.

### Study outcomes of clarithromycin in patients with stable CHD–PUD

The total follow-up summation was 15,180 person-years during the study period; the numbers of all-cause mortality, cardiovascular mortality, and cardiovascular morbidity were 794 (9.8%), 61 (0.7%), and 701 (8.6%), respectively (Table 2). The incidence rates of all-cause mortality, cardiovascular mortality, and cardiovascular morbidity were not high in clarithromycin users relative to nonusers. After adjustment for potential confounders listed in Table 1, the use of clarithromycin among patients with stable CHD–PUD was not significantly associated with an increased risk for all-cause mortality (adjusted hazard ratio [aHR], 0.93; 95% confidence interval [CI], 0.93–1.24), cardiovascular mortality (0.95; 0.57–1.59), and cardiovascular morbidity (0.94; 0.89–1.09).

**Table 2:** Risk of study outcomes comparing clarithormycin users versus nonusers

	Overall mortality			Cardiovascular mortality			Cardiovascular morbidity		
	Event	Incidence rate per 1000 patient-years	Adjusted HR* (95% CI)	Event	Incidence rate per 1000 patient-years	Adjusted HR* (95% CI)	Event	Incidence rate per 1000 patient-years	Adjusted HR^#^ (95% CI)
Nonusers (n = 4070)	399	21.2	1 (reference)	33	1.8	1 (reference)	360	20.2	1 (reference)
Clarithormycin users (n = 4070)	395	21.5	1.08 (0.93–1.24)	28	1.5	0.95 (0.57–1.59)	341	19.6	0.94 (0.89–1.09)

*HR* hazard ratio, *CI* confidence interval

*Adjusted for all covariates (age per year, sex, comorbidity, Charlson comorbidity index, number of medical visits, and drugs use) listed in Table 1

^#^Adjusted for all covariates (age per year, sex, comorbidity, Charlson comorbidity index, number of medical visits, and drugs use) listed in Table 1 and competing mortality

### Relationship between cumulative clarithromycin dose and study outcomes

There was no dose–response relationship between clarithromycin and overall mortality, cardiovascular mortality, and cardiovascular morbidity (Table 3).

**Table 3:** Adjusted hazard ratio (HR) of study outcomes associated with cumulative define daily dose (cDDD) of clarithromycin

Take nonusers as the reference	Overall mortality			Cardiovascular mortality			Cardiovascular morbidity		
	Events (n, %)	Adjusted HR* (95% CI)	*p* value	Events (n, %)	Adjusted HR* (95% CI)	*p* value	Events (n, %)	Adjusted HR^#^ (95% CI)	*p* value
cDDD of clarithromycin									
≦ 1000	25 (12.8)	1.68 (0.88–3.22)	0.12	1 (0.5)	Not converged	NA	12 (6.2)	1.00 (0.43–2.32)	0.99
> 1000–3000	66 (10.8)	2.33 (1.53–3.56)	< 0.0001	7 (1.1)	5.06 (0.78–32.74)	0.09	44 (7.2)	1.42 (0.87–2.32)	0.16
> 3000–5000	217 (9.2)	0.97 (0.81–1.17)	0.74	16 (0.7)	0.79 (0.41–1.54)	0.49	211 (9.0)	0.91 (0.75–1.10)	0.32
> 5000	87 (9.5)	0.89 (0.66–1.19)	0.42	4 (0.4)	0.63 (0.17–2.41)	0.50	74 (8.1)	0.83 (0.61–1.15)	0.27

*HR* hazard ratio, *CI* confidence interval

*Adjusted for all covariates (age per year, sex, comorbidity, Charlson comorbidity index, number of medical visits, and drugs use) listed in Table 1

^#^Adjusted for all covariates (age per year, sex, comorbidity, Charlson comorbidity index, number of medical visits, and drugs use) listed in Table 1 and competing mortality

### Relationship between the duration of clarithromycin prescription and study outcomes

There was no relationship between ≦ 7 days and > 7 days of clarithromycin prescription and overall mortality, cardiovascular mortality, and cardiovascular morbidity (Table 4).

**Table 4:** Adjusted hazard ratio (aHR) of study outcomes associated with the duration of clarithromycin prescription

	Overall mortality aHR* (95% CI)	Cardiovascular mortality aHR* (95% CI)	Cardiovascular morbidity aHR^#^ (95% CI)
Nonusers (n = 4070)	1 (reference)	1 (reference)	1 (reference)
≦ 7 days (n = 2273)	1.21 (1.02–1.43)	0.90 (0.50–1.62)	0.92 (0.77–1.10)
> 7 days (n = 1297)	0.81 (0.62–1.05)	1.13 (0.37–3.43)	0.99 (0.75–1.31)

*HR* hazard ratio, *CI* confidence interval

*Adjusted for all covariates (age per year, sex, comorbidity, Charlson comorbidity index, number of medical visits, and drugs use) listed in Table 1

^#^Adjusted for all covariates (age per year, sex, comorbidity, Charlson comorbidity index, number of medical visits, and drugs use) listed in Table 1 and competing mortality

### Relationship between clarithromycin and arrhythmia risk

Analysis of arrhythmia risk during follow-up period showed no increase in arrhythmia risk (aHR, 1.00; 95% CI, 0.90–1.10) among clarithromycin users, compared with nonusers (Table 5).

**Table 5:** Arrhythmia occurrence during follow-up

	Clarithromycin users (n = 4070)	Nonusers (n = 4070)
Events	791	800
Person-years observed	15,620	15,930
Incidence rate per 1000 person-years	50.7	50.2
Adjusted hazard ratio* (95% CI)	1.00 (0.90–1.10)	1 (reference)

*HR* hazard ratio, *CI* confidence interval

*Adjusted for all covariates (age per year, sex, comorbidity, Charlson comorbidity index, number of medical visits, and drugs use) listed in Table 1 and competing mortality

### Sensitivity analysis

After inclusion of arrhythmia events to re-estimate the risks of overall mortality (aHR, 1.08; 95% CI, 0.93–1.24, *p* = 0.31), cardiovascular mortality (aHR, 1.00; 95% CI, 0.62–1.63, *p* = 0.99), cardiovascular morbidity (aHR, 0.99; 95% CI, 0.91–1.08, *p* = 0.87), the result remained insignificant (Additional file 1: Table S1).

## Discussion

To our best knowledge, this is the first large nationwide cohort study using the new-user design and competing mortality and propensity score matching to demonstrate no increased risk for overall mortality, cardiovascular mortality, and cardiovascular morbidity in patients with stable CHD–PUD who were taking clarithromycin, compared with those who were not taking clarithromycin. We also found no dose–response relationship and dosing duration of clarithromycin with overall mortality and cardiovascular outcomes, and found no increased risk of arrhythmia associated with clarithromycin.

We selected stable CHD patients because we aimed to investigate the risk of clarithromycin in such a population similar to the CLARICOR trial [13]. We selected patients with PUD because clarithromycin could be used as an *H. pylori* eradication regimen after peptic ulcers are diagnosed. We did not identify other antibiotics in this study, such as amoxicillin or metronidazole, in combination with clarithromycin to analyze *H. pylori* eradication treatment as was performed by other studies [18, 19] because there are too many regimens (i.e., triple therapy, bismuth and non-bismuth quadruple therapy, sequential or concomitant therapy) that may have varying durations from seven to fourteen days [22, 34]. Besides, some patients may be allergic to penicillin antibiotics, so the regimens must be adjusted and individualized. Hence, if we consider all possibilities, the complexities may result in bias and complicated analyses. Therefore, we only considered clarithromycin use as an essential factor.

As was previously mentioned, peptic ulcers were considered primarily due to *H. pylori* infection. One study found that a subpopulation of patients with PUD had a lower CHD risk after *H. pylori* eradication [10]. However, this study excluded those with a history of CHD or patients taking antiplatelets before the index date upon evaluating the association of CHD with *H. pylori* eradication. Hence, the effects of anti-*H. pylori* antibiotics in patients who were previously diagnosed with CHD were unknown in that study. Our study found that overall mortality and cardiovascular mortality were not associated with clarithromycin use in patients with stable CHD and subsequent PUD. This result was similar to that in other studies. One study revealed neither risk nor benefit from clarithromycin use [18]. That study examined the risk of *H. pylori* eradication in patients with CHD, including a clarithromycin-containing or non-clarithromycin-containing regimen. It showed no mortality difference between clarithromycin-containing and non-clarithromycin-containing eradication treatments. Another study evaluated the cardiovascular events (myocardial infarction, stroke, angina, or transient ischemic attack) among those taking clarithromycin and other commonly used antibiotics to treat upper and lower respiratory tract infections. They found that the short-term cardiovascular risk looked no different between clarithromycin users and non-clarithromycin antibiotic users [35].

We also found that there was no relationship between clarithromycin and overall mortality and cardiovascular outcomes, regardless of the treatment doses or duration. No other studies investigating the relationship between heart events and the doses or duration of clarithromycin use except for one [14], but that study divided the treatment courses into < 3 days, 3–6 days, 7 days, and > 7 days. For *H. pylori* eradication therapy, the treatment course should be equal or more than seven days. Another study reporting more prescriptions of clarithromycin were associated with higher mortality risk [36], but they compared clarithromycin with other antibiotics such as doxycycline. The baseline characteristics in our clarithromycin users had higher aspirin intake, diabetes, and hypertension rates than nonusers. These differences implied that clarithromycin users in our patient population had more complicated diseases. However, they still could receive clarithromycin safely without increasing cardiovascular morbidities. Other studies showed that clarithromycin has no association with arrhythmia [37] or stroke [19, 38]. In the study of a 10-year follow-up of the CLARICOR trial, Winkel et al*.* found that an increased risk of stroke only occurred in nonusers of statin [39]. Moreover, Winkel et al*.* found that the previously mentioned adverse effect from clarithromycin was reversed during the last 4 follow-up years. That is, clarithromycin users had significantly lower cardiovascular death. Statin still played a protective role in those patients. Statin users had much lower all-cause mortality than those not on a statin. Besides, among those statin users, all-cause mortality, cardiovascular mortality, and cerebrovascular events were not significantly different between the clarithromycin and placebo groups during the 10-year follow-up period. We may also interpret that statin is protective in those patients with CHD who did and did not take clarithromycin. In another study, Jensen et al. further analyzed the subgroup in the CLARICOR trial. They reported that statin users in the clarithromycin group had no increased cardiovascular mortality compared with those in the placebo group [40]. In our study, we did not specifically analyze the effect of statin because we have viewed statin as a possible confounding factor in multivariable analysis. Besides, we performed propensity score matching in our study. In the past, clinicians did not pay as much attention to the importance of statin as clinicians do presently. According to current practice guidelines, most patients with CHD should take statin as a primary or secondary preventative treatment [41]. Hence, if clarithromycin has a cardiovascular risk, statin may partly offset that risk in patients with CHD.

Svanström et al. also found that clarithromycin was related to a higher rate of cardiac death when compared with roxithromycin and penicillin V after 7-day treatments [42]. However, the incidence of cardiac death from clarithromycin was mildly higher than that of penicillin V (5.3 per 1000 person years and 2.5 per 1000 person years, respectively). Furthermore, Inghammar et al. analyzed the use of clarithromycin, roxithromycin, and penicillin V after treatment was started. They found that the cardiac risk from clarithromycin seemed to occur during current use, but later, the adverse effect was reduced after a more extended period of follow-up [43]. Wong et al. evaluated the cardiovascular risk from clarithromycin use in the general population. They also found that the current use of clarithromycin for *H. pylori* eradication was associated with myocardial infarction and arrhythmia; however, the adverse effects wore off after the treatment course of clarithromycin [19]. In contrast with Wong et al.'s conclusions, the CLARICOR trial [13] revealed no significant differences in all-cause mortality, myocardial infarction, or unstable angina pectoris between clarithromycin users and placebo users when they followed up those participants in the first month. Therefore, whether the current use of clarithromycin increases cardiovascular risk is controversial. The exact mechanism accounting for possible excess cardiac mortality is not fully understood. Antibiotics-associated QT interval prolongation may be a key point. Such antibiotics include macrolides, fluoroquinolones, antimalarials, etc. They may delay the ventricular repolarization and lead to QT prolongation, which possibly further induces *torsades de pointes* [44]. To realize the arrhythmia-associated outcomes in our population, we analyzed the risk of arrhythmia occurrence between clarithromycin users and nonusers. We further evaluated the role of arrhythmia in overall mortality, cardiovascular mortality, and cardiovascular morbidity. Our analyses showed the risks of arrhythmia and arrhythmia-associated outcomes were not different between clarithromycin users and nonusers. Clarithromycin alone may be safe in patients with stable CHD, but concomitant use of medications believed to prolong the QT interval may substantially increase cardiac risks [45]. So we should follow up these patients during the treatment course or avoid clarithromycin-based *H. pylori* eradication regimens if concomitant risk factors for QT interval prolongation exist.

Our CHD–PUD population accounted for 15% of CHD population in 2001–2015. The NHI program adopts ICD-9-CM diagnosis codes to define diseases and we identified PUD patients by ICD-9-CM codes 531–534, the method of which was used in prior NHIRD-based research [27, 46]. A hospital-based study in Taiwan showed that the prevalence of PUD confirmed by gastroendoscopy was 9.4% in asymptomatic subjects from January to August 2008 [47]. Another NHIRD-based study showed the PUD population accounted for 25.5% of general population in 2000–2013 [46]. Convenient accessibility of medical care and easy availability to gastroendoscopy in Taiwan might account for the high prevalence of PUD.

By analyzing the NHI claims data with a highly representative sample, the present study has five strengths. First, recall bias of clarithromycin was avoided. Second, using the new-user design minimized the immortal bias and the potential residual effect of using clarithromycin before CHD inception date. Third, the follow-up of death and cardiovascular outcomes was complete, and the use of competing mortality minimized risk overestimation of cardiovascular morbidity. Fourth, the use of propensity score matching minimized confounding effects. Fifth, consideration of medical services minimized detection bias [31]. However, several potential limitations exist. First, the NHI claims data lack information on lifestyle (*e.g.*, smoking, alcohol consumption, diet, and physical activity), body weight, levels of blood pressure and sugar, the eradication rate of *H. pylori*, and laboratory data (*e.g. H. pylori* virulence factor such as cytotoxin-associated gene A), which may contribute to the risks of death and cardiovascular outcomes. Nevertheless, we added the CCI score into the propensity analysis to reach the comparability of both cohorts. We included the CCI score in multivariable analyses to control confounding factors in healthcare administrative databases [29, 31]. Second, the compliance of prescribed clarithromycin was not assessed in the administrative claims. Third, the information on self-paid clarithromycin was not evaluated in the administrative claims. However, it seems to have rarely occurred because the antibiotic prescription was under strict regulations of the NHI program. Fourth, this was a retrospective study instead of a prospective study. However, it was not ethical to conduct a study where patients with stable CHD were diagnosed with acute infection and clarithromycin was indicated, but one group took the proper medication and the other took a placebo. Finally, unmeasured confounders may still exist as in any observational study.

## Conclusion

This national cohort study indicates that clarithromycin is not associated with an increased risk of overall mortality, cardiovascular mortality, and cardiovascular morbidity in patients with stable CHD and subsequent PUD. This seems to be important for gastroenterologists when clarithromycin-containing *H. pylori* eradication therapy is considered.

## Supplementary Information

**Additional file 1.** **Supplemental Table 1.** Risk of study outcomes including arrhythmia events comparing clarithormycin users vs. nonusers.

## Data Availability

Data are available from the Taiwan's 2005 Longitudinal Generation Tracking Database (LGTD2005) published by the Health and Welfare Data Science Center (HWDSC). The datasets utilized in this study cannot be made available in the paper, the additional files, or in a public repository due to the "Personal Information Protection Act" executed by Taiwan’s government, starting from 2012. Interested researchers can obtain the sorting data from the corresponding author on reasonable request after the HWDSC's agreement.

## Abbreviations

*H. pylori*: *Helicobacter pylori*
CHD: Coronary heart disease
COPD: Chronic obstructive pulmonary disease
LGTD: Longitudinal Generation Tracking Database
NHI: National Health Insurance
PAOD: Peripheral arterial occlusive disease
CCI: Charlson comorbidity index
ACEI: Angiotensin-converting enzyme inhibitor
ARB: Angiotensin II receptor antagonist
