# Supplementary material for: A nationwide cohort study suggests clarithromycin-based therapy for Helicobacter pylori eradication is safe in patients with stable coronary heart disease and subsequent peptic ulcer disease
Source: BMC Gastroenterol. 2022 Sep 12;22:416. doi: 10.1186/s12876-022-02498-1 (PMC9469559; doi:10.1186/s12876-022-02498-1)
Supplement: Supplementary file 1 — Additional file 1. Supplemental Table 1. Risk of study outcomes including arrhythmia events comparing clarithormycin users vs. nonusers. [file 12876_2022_2498_MOESM1_ESM.doc]

| **Supplemental Table 1.** Risk of study outcomes including arrhythmia events comparing clarithormycin users *vs.* nonusers | | | | | | | | | | | |
| --- | --- | --- | --- | --- | --- | --- | --- | --- | --- | --- | --- |
|  | Overall mortality | | |  | Cardiovascular mortality | | |  | Cardiovascular morbidity | | |
|  | Event | Adjusted HR^*^  (95% CI) | *p*-value |  | Event | Adjusted HR^*^  (95% CI) | *p*-value |  | Event | Adjusted HR^#^  (95% CI) | *p*-value |
| Nonusers  (n=4,070) | 399 | 1 (Reference) |  |  | 36 | 1 (Reference) |  |  | 1011 | 1 (Reference) |  |
| Clarithormycin users (n=4,070) | 395 | 1.08 (0.93-1.24) | 0.31 |  | 32 | 1.00 (0.62-1.63) | 0.99 |  | 1000 | 0.99 (0.91-1.08) | 0.87 |
| Abbreviations: HR, hazard ratio; CI, confidence interval.  ^*^Adjusted for all covariates (age per year, sex, comorbidity, Charlson comorbidity index, number of medical visits, and drugs use) listed in Table 1.  ^#^Adjusted for all covariates (age per year, sex, comorbidity, Charlson comorbidity index, number of medical visits, and drugs use) listed in Table 1 and competing mortality. | | | | | | | | | | | |
